# Serum α-hydroxybutyrate dehydrogenase as a biomarker for predicting survival outcomes in patients with UTUC after radical nephroureterectomy

**DOI:** 10.1186/s12894-024-01439-2

**Published:** 2024-03-20

**Authors:** Jianjun Ye, Lei Zheng, Zeyu Chen, Qihao Wang, Xinyang Liao, Xingyuan Wang, Qiang Wei, Yige Bao

**Affiliations:** 1 Department of Urology and Institute of Urology, West China Hospital, Sichuan University, Chengdu, 610041 China; 2 West China School of Medicine, Sichuan University, Chengdu, China; 3 Department of Urology, People’s Hospital of Tibet Autonomous Region, Lhasa, China

**Keywords:** α-hydroxybutyrate dehydrogenase, Upper tract urothelial carcinoma, Radical nephroureterectomy, Biomarker, Outcome

## Abstract

**Purpose:**

We aimed to determine the prognostic value of α-hydroxybutyrate dehydrogenase (α-HBDH) in upper tract urothelial carcinoma (UTUC) patients after radical nephroureterectomy (RNU).

**Materials and methods:**

We retrospectively enrolled the data of 544 UTUC patients at West China Hospital from May 2003 to June 2019. Cancer-specific survival (CSS) was the endpoint of interest. The optimal cutoff value of α-HBDH was identified by X-Tile program. After propensity score matching (PSM), we utilized Kaplan‒Meier curves to estimate survival and Cox proportional hazard model for risk assessment. A nomogram was built based on the results of multivariate analysis, and calibration curve, time-dependent receiver operating characteristic (ROC) curves and decision curve analysis were also performed to evaluate the predictive accuracy.

**Results:**

Overall, 394 and 150 patients were divided into the α-HBDH-low group and α-HBDH -high group at the cutoff value of 158 U/L, respectively. After PSM, the two groups were well matched for all confounding factors. High α-HBDH was associated with inferior CSS (*P* = 0.006), and preoperative α-HBDH was an independent predictor for CSS (HR: 1.36; 95% CI:1.08, 1.80), especially in localized UTUC patients (HR: 2.04; 95% CI:1.11, 3.74). Furthermore, the nomogram based on α-HBDH achieved great predictive ability for CSS with areas under the curves of 0.800 and 0.778 for 3-year and 5-year CSS, respectively.

**Conclusion:**

Serum α-HBDH was a novel and reliable biomarker for predicting survival outcomes in UTUC patients after RNU but should be further explored.

**Supplementary Information:**

The online version contains supplementary material available at 10.1186/s12894-024-01439-2.

## Introduction

Upper urinary tract urothelial carcinoma (UTUC) is an uncommon disease, accounting for 5–10% of urothelial carcinomas, and has an estimated annual incidence of approximately 2 people per 100,000 [1, 2]. Radical nephroureterectomy (RNU) remains the gold standard treatment for high-risk organ-confined UTUC, while nephron-sparing surgery might be an appropriate choice for patients with low-risk disease and patients with solitary kidney or severe renal impairment. Overall, two-thirds of UTUCs are invasive at first diagnosis, and the 5-year cancer-specific survival ranges from 30 to 60%, which places a great health and financial burden on individuals and countries [3, 4].

Over the past decades, precision or personalized medicine approaches have attracted much attention and have successfully accelerated the rapid development of the novel therapies and prognostic prediction [5]. In addition to the routine clinicopathological variables, emerging nutritional or inflammation-related indexes, such as the neutrophil lymphocyte ratio and systemic immune-inflammation index, were proven to be independent factors of survival outcomes [6, 7]. Our center also focused on the relevant factors and reported the prognostic role of lactate dehydrogenase (LDH) in UTUC patients in 2019 [8]. Serum α-hydroxybutyrate dehydrogenase (α-HBDH), an isoenzyme of LDH, was used as an indicator of myocardial damage and was recently found to be increased in patients with malignant tumors of the lung, testis and hematology [9–11]. In Yuan’s study, α-HBDH even had a higher sensitivity than LDH in predicting the prognosis of lung cancer patients [11]. However, whether there are significant changes in serum α-HBDH in UTUC patients and whether they have predictive value for survival outcomes have not been reported.

Therefore, the aim of the present study was to determine the prognostic value of α-HBDH in UTUC patients in Southwest China by using a relatively large cohort.

## Methods and materials

### Patients and data

Our study was approved by the Ethics Committee on Biomedical Research, West China Hospital of Sichuan University (2021–1209), and due to the retrospective design, anonymous data and confidential information of the included patients, this study was allowed to be conducted under waiver of informed consent by the local institutional review board.

We retrospectively collected patients with UTUC who underwent RNU between May 2003 and June 2019 at West China Hospital of Sichuan University. Patients with incomplete long-term prognostic information, patients without records of serum α-HBDH, patients who underwent neoadjuvant therapy, and patients with pathologically confirmed nonurothelial carcinoma were excluded from the present study. Finally, 544 patients were taken for further study.

Laboratory data within 3 days before surgery and clinicopathologic features of the included patients were extracted from medical records. Laboratory data included LDH and α-HBDH. Clinicopathologic features included chronological age in the year of surgery, gender, body mass index (BMI), tumor characteristics (including location, size, pathological stage, grade, architecture and necrosis), lymphovascular invasion (LVI), lymph node status, surgical margin and adjuvant systemic chemotherapy.

The specimens obtained by RNU were independently reviewed by our professional genitourinary pathologists. Then histopathological staging was conducted in accordance with the American Joint Committee on Cancer (AJCC) tumor-node-metastasis (TNM) staging system, and histopathological grading was performed according to the WHO/ISUP recommendation grading system.

### Follow-up and outcomes

The first follow-up was conducted 2 to 3 weeks after RNU when the pathology report of the surgery specimen had been completed. The main project of the first follow-up was to analyze the characteristics of the primary tumor and make subsequent therapy plans. In general, the follow-up protocol was in concordance with the European Association of Urology (EAU) guidelines. The follow-up interval was every 3 months in the first year after RNU, every 6 months in the second year, and a year for the rest of the time if there was no sign of recurrence or any uncommon symptoms. The physical examination, laboratory tests including blood and urine, contrast-enhanced computed tomography (CT) scan of chest and abdomen, and cystoscopy were the routine follow-up contents.

Cancer-specific survival (CSS) and the corresponding events (cancer-specific mortality (CSM)) were included as the end points of interest. CSS was defined as the period from RNU to death owing to UTUC.

### Statistical analysis

Data were analyzed with the use of the statistical package R (Foundation: http://www.r-projiect.org; version 4.1.3) and Empower(R) (Foundation: http://www.empowerstats.com). X-Tile 3.6.1 software (Yale University) was used to identify the best cut point of α-HBDH, LDH.

and age based on the lowest *P* values and the maximum chi-square of log-rank tests [12].

Continuous variables were described based on medians and interquartile ranges (IQRs), and categorical variables were described based on numbers and percentages. Student’s t test or the Mann–Whitney U test as well as the chi-square or Fisher’s exact test were performed to compare significant differences in clinicopathological characteristics. Propensity score matching (PSM), according to tumor stage, tumor grade and lymph node status, with one-to-two pair matching was used to balance differences caused by potential confounders. CSS was calculated using Kaplan–Meier method and compared using the log-rank test. Cox proportional hazard models were used to investigate the associations between preoperative α-HBDH and survival outcomes by hazard ratios (HRs) and 95% confidence intervals (95% CI). Every variable with a *P* value of less than 0.1 in the univariate Cox regression was included in the multivariate Cox regression for further analysis. Additional subgroup analyses were performed according to pathological tumor stage.

Furthermore, those variables with *P* values less than 0.05 in multivariate analyses were utilized to construct a nomogram for 3-year and 5-year CSS prediction. Time-dependent receiver operating characteristic (ROC) curves, calibration curves and decision curve analysis (DCA) were used to evaluate the performance of the nomogram. All results of the analysis were considered to be statistically significant with a two-sided *P* < 0.05.

## Results

### Basic characteristics in total and grouped patients

A total of 544 UTUC patients who had undergone RNU were enrolled in our study, with a male to female ratio of 303:241 and a median age of 68 years. A total of 272 (50.28%) patients were diagnosed with localized UTUC (Ta/Tis/T1/T2) and 269 (49.72%) patients had locally advanced UTUC (T3/T4). Tumor grades were distributed as low in 129 (23.89%) and high in 411(76.11%) cases. The final pathological reports showed that LNM, LVI, tumor necrosis, papillary architecture and positive surgery margin occurred in 55 (10.15%), 87 (16.08%), 45 (8.27%), 291 (54.19%) and 43 (7.95%) cases, respectively.

Among all patients, the mean value of α-HBDH was 139.5 U/L. By using X-Tile 3.6.1 software, the best cutoff point of α-HBDH was identified based on the lowest *P* values and the maximum chi-square of log-rank tests, and the optimal cutoff value was determined to be 158 U/L (Supplementary Fig. 1). Therefore, 394 (72.43%) patients were categorized into the α-HBDH-low group and the remaining 150 (27.57%) patients were assigned to the α-HBDH-high group. Significant differences were observed between the α-HBDH-high group and α-HBDH-low groups in terms of the gender (*P* < 0.001), LDH (*P* < 0.001), tumor location (*P* = 0.007), lymph node status (*P* = 0.005) and tumor necrosis (*p* < 0.001).

After PSM for tumor stage, tumor grade and lymph node status, we derived 150 paired cohorts at a ratio of 1 to 2 (according to the high/low value of α-HBDH). These two groups were well-matched for all confounding variables except for LDH (*P* < 0.001).

Detailed characteristics are given in Table 1.

**Table 1:** The clinical and pathological characteristics of ungrouped, and grouped patients before or after PSM

Variables	Total patients (*n* = 544)	Before PSM			After PSM^a^		
		α-HBDH-low group (*n* = 394)	α-HBDH-high group (*n* = 150)	*P* value	α-HBDH-low group (*n* = 300)	α-HBDH-high group (*n* = 150)	*P* value
Age (IQR)	68.00 (61.00–75.00)	68.00 (61.00–75.00)	69.00 (64.00–75.00)	0.623	68.00 (61.00–75.00)	69.00 (64.00–75.00)	0.623
Age, n (%)				0.052			0.079
< 65	191 (35.11%)	148 (37.56%)	43 (28.67%)		111 (37.00%)	43 (28.67%)	
≥ 65	353 (64.89%)	246 (62.44%)	107 (71.33%)		189 (63.00%)	107 (71.33%)	
Body mass index (IQR)	22.67 (20.76–25.35)	22.66 (20.77–25.43)	22.76 (19.90–24.35)	0.490	22.66 (20.77–25.43)	22.70 (19.90–24.35)	0.710
α-HBDH (IQR)	139.50 (122.75–161.00)	131.00 (119.00–142.00)	175.50 (164.25–203.50)	< 0.001	133.00 (119.80–143.00)	175.50 (164.20–203.50)	< 0.001
LDH (IQR)	179.00 (157.75–202.00)	168.00 (151.00–182.00)	222.50 (205.00–251.75)	< 0.001	169.00 (150.80–182.20)	222.50 (205.00–251.80)	
LDH, n (%)				< 0.001			< 0.001
≤ 200	401 (73.71%)	372 (94.42%)	29 (19.33%)		286 (95.33%)	29 (19.33%)	
> 200	143 (26.29)	22 (5.58%)	121 (80.67%)		14 (4.67%)	121 (80.67%)	
Albumin (IQR)	40.55 (37.00–43.10)	40.80 (37.80–43.30)	39.60 (35.00–43.00)	0.025	40.20 (38.20–43.20)	39.60 (35.00–43.00)	0.058
Gender, n (%)				< 0.001			0.124
Female	241 (44.30%)	153 (38.83%)	88 (58.67%)		153 (51.00%)	88 (58.67%)	
Male	303 (55.70%)	241 (61.17%)	62 (41.33%)		147 (49.00%)	62 (41.43%)	
Tumor location, n (%)				0.007			0.514
Renal pelvis	271 (49.91%)	190 (48.35%)	81 (54.00%)		170 (56.67%)	81 (54.00%)	
Ureter	209 (38.49%)	165 (41.98%)	44 (29.33%)		92 (30.67%)	44 (29.33%)	
Both	63 (11.60%)	38 (9.67%)	25 (16.67%)		38 (12.67%)	25 (16.67%)	
Tumor size, n (%)				0.392			0.325
< 3	186 (34.38%)	139 (35.46%)	47 (31.54%)		108 (36.24%)	47 (31.54%)	
≥ 3	355 (65.62%)	253 (64.54%)	102 (68.46%)		190 (63.76%)	102 (68.46%)	
Tumor stage, n (%)				0.128			0.461
≤ pT2	272 (50.28%)	205 (52.30%)	67 (44.97%)		145 (48.66%)	67 (44.97%)	
> pT2	269 (49.72%)	187 (47.70%)	82 (55.03%)		153 (51.34%)	82 (55.03%)	
Tumor grade, n (%)				0.052			0.354
Low	129 (23.89%)	102 (26.09%)	27 (18.12%)		65 (21.89%)	27 (18.12%)	
High	411 (76.11%)	289 (73.91%)	122 (81.88%)		232 (78.11%)	122 (81.88%)	
Lymph node status, n (%)				0.005			0.084
pN0/x	487 (89.85%)	362 (92.11%)	125 (83.89%)		269 (89.67%)	126 (84.00%)	
pN +	55 (10.15%)	31 (7.89%)	24 (16.11%)		31 (10.33%)	24 (16.00%)	
Lymphovascular invasion, n (%)				0.065			0.156
Yes	87 (16.08%)	56 (14.29%)	31 (20.81%)		46 (15.44%)	31 (20.81%)	
No	454 (83.92%)	336 (85.71%)	118 (79.19%)		252 (84.56%)	118 (79.19%)	
Tumor architecture, n (%)				0.290			0.584
Sessile	246 (45.81%)	185 (47.19%)	61 (42.07%)		134 (44.82%)	61 (42.07%)	
Papillary	291 (54.19%)	207 (52.81%)	84 (57.93%)		165 (55.18%)	84 (57.93%)	
Surgery margin, n (%)				0.139			0.077
Positive	43 (7.95%)	27 (6.89%)	16 (10.74%)		18 (6.04%)	16 (10.74%)	
Negative	498 (92.05%)	365 (93.11%)	133 (89.26%)		280 (94.96%)	133 (89.26%)	
Tumor necrosis, n (%)				0.891			0.487
Yes	92 (16.91%)	67 (17.01%)	25 (16.67%)		42 (14.00%)	25 (16.67%)	
No	452 (83.09%)	327 (82.99%)	125 (83.33%)		258 (86.00%)	125 (83.33%)	
Adjuvant systemic chemotherapy, n (%)				0.265			0.708
Yes	84 (14.14%)	55 (14.00%)	29 (19.32%)		45 (15.00%)	29 (19.32%)	
No	460 (85.86%)	339 (86.00%)	121 (80.68%)		255 (85.00%)	121 (80.68%)	

*Abbreviations*: *IQR* Interquartile range, *α-HBDH* α-hydroxybutyrate dehydrogenase, *LDH* Lactate dehydrogenase, *PSM* Propensity score matching

^a^Propensity score matching was used according to tumor location, gender and lymph node status, with one-to-two pair matching to balance differences caused by potential confounders

### Survival outcomes

**Fig. 1:**
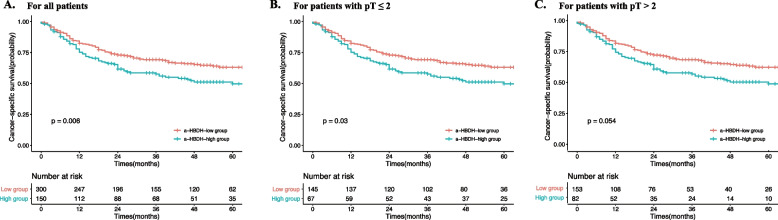
Kaplan–Meier curves for cancer-specific survival stratified by α − HBDH. Abbreviations: α-HBDH = α-hydroxybutyrate dehydrogenase

The median follow-up time was 59 months (IQR: 32–83). During this period, 201 (36.9%) patients had died from UTUC, and the 5-year CSS rates were 54.3% and 67.9% for patients in the α-HBDH-high group and α-HBDH-low group, respectively. The Kaplan‒Meier plots showed that, compared with patients in the α-HBDH-low group, patients in the α-HBDH-high group tended to have significantly poorer CSS (*P* = 0.006) (Fig. 1A). Furthermore, subgroup analysis was performed on pathological tumor stage, and a significant difference in CSS between the α-HBDH-low group and the α-HBDH-high group was observed only in localized UTUC patients (pT ≤ 2) (*P* = 0.030) (Fig. 1B), but not in locally advanced UTUC patients (*P* = 0.054) (Fig. 1C).

### Univariate and multivariate cox regression

The results of univariate and multivariate Cox regression about the prognostic value of α-HBDH for CSS are shown in the Supplementary Table 1. To include as many clinical indicators as possible, we included all variables whose *P* values were less than 0.1 in the univariate Cox regression into the multivariate Cox regression. The results demonstrated that age (HR: 0.62, 95% CI: 0.45, 0.85), serum α-HBDH (HR: 1.36, 95% CI: 1.08, 1.80), tumor size (HR: 1.44, 95% CI: 1.01, 2.06), tumor stage (HR: 2.42, 95% CI: 1.62, 3.61), tumor grade (HR: 1.98, 95% CI: 1.17, 3.37) and lymph node status (HR: 2.17, 95% CI: 1.47, 3.19) were independent predictors for CSS (Table 2) (Supplementary Table 1). Cox proportional hazard models were also utilized in the subgroup analysis of pathological tumor stage. The results indicated that, serum α-HBDH was an independent risk factor for CSS in localized UTUC patients (HR: 2.01, 95% CI: 1.11, 3.74) (pT ≤ 2), but not in locally advanced UTUC patients (HR: 1.00, 95% CI: 0.62, 1.61) (pT > 2) (Table 2) (Supplementary Table 2 and Supplementary Table 3).

**Table 2:** The results of univariate and multivariate analysis about α-HBDH on cancer-specific survival

Groups	Cancer-specific survival	
	Univariate	Multivariate
	HR (95%CI), P	HR (95%CI), P
All UTUC patients		
α-HBDH-low group	Reference	Reference
α-HBDH-high group	1.53 (1.13, 2.07) 0.006	1.36 (1.08, 1.80) 0.034
Patients with pT ≤ 2		
α-HBDH-low group	Reference	Reference
α-HBDH-high group	1.92 (1.05, 3.51) 0.033	2.04 (1.11, 3.74) 0.021
Patients with pT > 2		
α-HBDH-low group	Reference	Reference
α-HBDH-high group	1.41 (0.99, 2.01) 0.059	1.00 (0.62, 1.61) 0.994

The detailed results of univariate and multivariate analysis were shown in the supplementary files. Variables included in multivariate analysis were extracted from variables with *P* value of less than 0.1 in univariate analysis

*Abbreviations*: *HR* Hazard ratio, *CI* Confidence interval, *α-HBDH* α-hydroxybutyrate dehydrogenase, *UTUC* Upper tract urothelial carcinoma

### Nomogram construction and validation

**Fig. 2:**
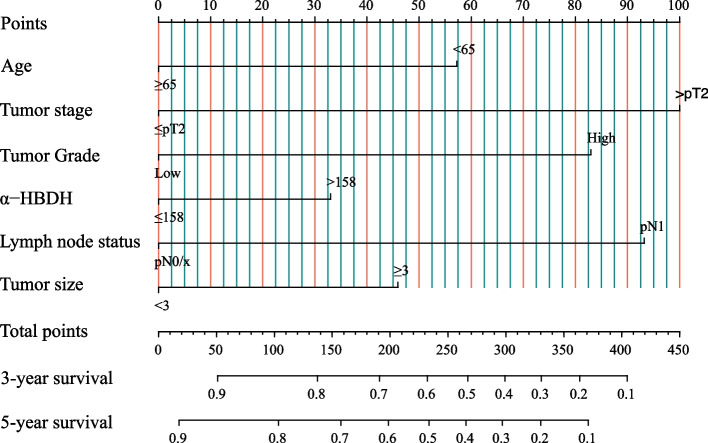
A nomogram for predicting 3-year or 5-year cancer-specific survival

**Fig. 3:**
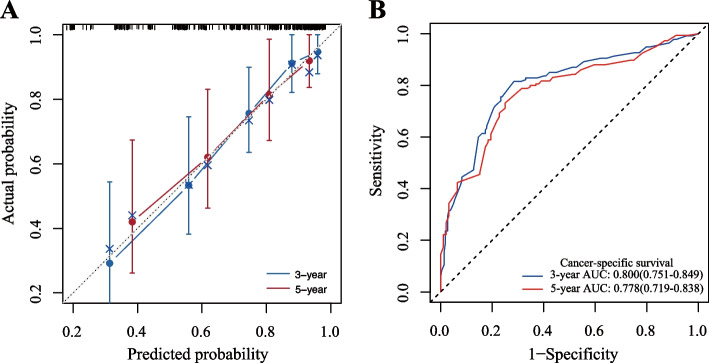
Calibration curves of 3-year and 5-year cancer-specific survival (**a**) and ROC curve for assessing the calibration and discrimination of the nomogram in predicting 3-year or 5-year cancer-specific survival (**b**)

**Fig. 4:**
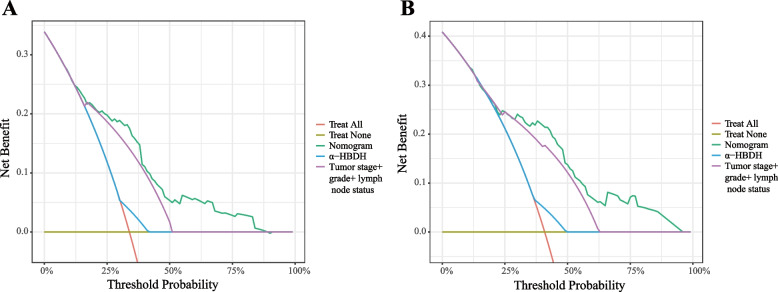
Decision curve analysis of the nomogram about 3-year cancer-specific survival benefit (**A**) and 5-year cancer-specific survival benefit (**B**)

Then, based on the results of multivariate Cox analysis, 6 variables were finally included in the nomogram: age, pathological tumor stage, tumor grade, α-HBDH, lymph node status and tumor size for predicting the 3-year and 5-year CSS of UTUC patients after RNU (Fig. 2). The calibration curves revealed high consistencies between the predicted and observed survival probabilities and responsible reproducibility (Fig. 3A). Time-dependent ROC curves were further utilized to evaluate the prediction accuracies of the nomograms and the results indicated that the nomograms had sound performance in predicting CSS with AUCs of 0.800 and 0.778 for 3-year CSS and 5-year CSS, respectively (Fig. 3B). Moreover, DCA curves showed that the nomogram could better predict the 3‐year and 5‐year CSS, as it added more net benefits compared with the conventional models consisting of pathological tumor stage, tumor grade and lymph node status (Fig. 4).

## Discussion

As the first study to evaluate the prognostic value of serum α-HBDH in UTUC patients after RNU, the present study proposed that preoperative serum α-HBDH value was an independent biomarker for predicting survival outcomes by retrospectively analyzing 544 UTUC patients who underwent RNU in Southwest China. Moreover, we built a nomogram based on α-HBDH and 5 other variables, and verified the predictive accuracy of the nomogram to further evaluate the predictive value of α-HBDH.

α-HBDH, which plays a role in catalyzing the oxidation of α-hydroxybutyrate to α-ketobutyric acid, is one of the enzymes that contributes to the formation of the myocardial enzyme spectrum and reflects hemolysis and heart injury when the level increases [13, 14]. The increase in α-HBDH in serum was not only the result of myocardial injury, but also associated with the damage to the renal and hematologic systems, which reflected the abnormity of multiple systems and might be a predictive factor for adverse prognosis of patients with systemic disease, such as viral infection and malignant carcinomas.

In 2021, Liu et al. reported their findings that the in-hospital mortality and disease severity of patients infected by SARS‐Cov‐2 were significantly associated with the α-HBDH level [15, 16]. However, such findings were confounded by the coexisting factors (age, white blood cell count, IL‐6, D‐dimer, LDH, lymphocyte count, and albumin levels), and further research about the specific mechanism is needed. In the present study, age, albumin level and LDH were also included. Although UTUC patients in the α-HBDH-high group had advanced age and lower albumin levels, these two factors did not have a significant impact on oncological outcomes. Our data showed that α-HBDH, which is an isoenzyme of LDH and could represent LDH1 and LDH2, has a similar or parallel expression with LDH in serum [17]. However, further Cox analysis indicated that there was a significant association between the serum α-HBDH value and CSS in the whole group and even localized UTUC, while such an association was not found for LDH. Interestingly, a study, conducted by our center, investigated the effect of LDH on the prognosis of UTUC and found that the LDH-high group did not reach a significant difference in metastasis-free survival with the comparison of LDH-low group [8]. The different cutoff values of LDH might be the reasons for such a difference. More future studies are needed to validate the conclusion and further explore the relationship by combining LDH and α-HBDH.

Previous studies on α-HBDH have mostly focused on myocardial infarction, atherothrombotic and liver injury and only a few on cancers [14, 18, 19]. The phenomena concerning the elevation of serum α-HBDH in malignant carcinomas have been observed in several specific cancers. In children, the simultaneous presence of high α-HBDH values with musculoskeletal symptoms suggests the necessity of screening for the occult tumors, such as acute lymphocytic leukemia, lymphoma, neuroblastoma and so on [9]. After comparing 51 cases of testicular germ tell tumors with 40 healthy controls, Khanolkar et al. proposed that serum LDH and α-HBDH could both be used as tumor markers in the diagnosis of testicular germ cell tumors as well as prognostic indicators in monitoring therapy. Moreover, α-HBDH was more specific in monitoring therapy than serum LDH [10]. Recently, Yuan et al. launched a study on the prognostic value of α-HBDH in lung cancer patients, and found that α-HBDH was an independent risk factor for CSS and that the sensitivity of α-HBDH was higher than that of LDH [11]. Although the cutoff value of α-HBDH in our study (158 U/L) was different from that in Yuan’s study (220 U/L), we divided the included patients at a ratio of nearly 1:3. Moreover, benefitting from a relatively larger sample size, in the present study, we further performed subgroup analysis by pathological tumor stage and found a more precise conclusion, which might represent precise applicability. Therefore, the application of α-HBDH for oncological outcome prediction is only in its infancy, and whether the ability of α-HBDH to predict survival is effective for other tumors remains to be witnessed.

In addition to the tumor size, stage and grade which are level C evidence recommended by the EAU guidelines, another independent risk factor, that cannot be ignored, for CSS is the lymph node status [2]. In our study, patients with LNM tended to be associated with worse CSS, which was consistent with a systematic review [20]. However, there were several adverse conditions that might affect the conclusion. In our center, lymphadenectomy was not performed routinely (72.3% patients were not staged with a lymph node dissection in our study) and only for patients with abnormal examination about lymph nodes preoperatively or palpable lymph nodes intraoperatively, which meant that the positive rate of lymph nodes might be overestimated. What’s more, we regarded patients who were lymph node-negative and unevaluated as the negative group, which would reduce the proportion of patients in the lymph node-positive group and thus render the inauthentic effect of node-positivity on prognosis.

In the present study, albumin was not an independent risk factor for CSS. Hypoalbuminemia was previously proven to be strongly associated with cancer patients’ worsened OS and PFS, but the clinical practice of the single factor was limited because it could be easily affected by liver function, fluid volume and systemic inflammation [21–23]. This might be the reason for the results in our study. Recently, based on the level of albumin, the novel nutrition assessment tools, named the Prognostic Nutrition Index (PNI) and Naples prognostic score (NPS), have gained increasing popularity in predicting the prognosis of patients with different cancers [24, 25]. Data from our center showed that NPS and PNI were easy and effective tools in predicting OS in UTUC patients especially in locally advanced patients, which emphasized the importance of the nutrition status of patients with malignant carcinomas and advocated paying more attention to managing nutrition status throughout cancer treatment.

Nonetheless, our study had some limitations. First, it was performed in a retrospective, single-center designed study, which might lead to selection bias and be different from other studies’ findings. Second, the cutoff value of α-HBDH came from X-Tile software, which was different from that in other studies. The applicability of the conclusion is limited. Third, the information about comorbidities of UTUC patients before or at the time of RNU was not recorded, and coexisting disease, such as myocardial infarction, atherothrombotic and liver injury, might affect the α-HBDH level and further impact the conclusion. We are conducting a prospective, multicenter study with more improved baseline data to address these limitations.

## Conclusion

In summary, high α-HBDH tended to be associated with inferior CSS, and preoperative α-HBDH is an independent biomarker for predicting survival outcomes in UTUC patients after RNU. The nomogram including α-HBDH is a promising personalization tool to provide reliable prognostic information for achieving the greatest survival benefits in UTUC patients, but further external validation is needed.

### Supplementary Information

**Supplementary Material 1.**

## Data Availability

The data that support the findings of this study are available on request from the corresponding author, upon reasonable request.

## Abbreviations

UTUC: Upper tract urothelial carcinoma
RNU: Radical nephroureterectomy
CSS: Cancer-specific survival
PSM: Propensity score matching
ROC: Receiver operating characteristic
α-HBDH: α-Hydroxybutyrate dehydrogenase
LDH: Lactate dehydrogenase
BMI: Body mass index
LVI: Lymphovascular invasion
AJCC: American Joint Committee on Cancer
TNM: Tumor-node-metastasis
CT: Computed tomography
EAU: European Association of Urology
CSM: Cancer-specific mortality
HRs: Hazard ratios
95% CI: 95% Confidence intervals
DCA: Decision curve analysis
PNI: Prognostic Nutrition Index
NPS: Naples prognostic score
