# Supplementary material for: Serum α-hydroxybutyrate dehydrogenase as a biomarker for predicting survival outcomes in patients with UTUC after radical nephroureterectomy
Source: BMC Urol. 2024 Mar 20;24:62. doi: 10.1186/s12894-024-01439-2 (PMC10953183; doi:10.1186/s12894-024-01439-2)
Supplement: Supplementary file 1 — Supplementary Material 1. [file 12894_2024_1439_MOESM1_ESM.docx]

**Supplementary Table 1.** The results of univariate and multivariate analysis about α-HBDH on cancer-specific survival in the whole group.

| Variables | Cancer-specific survival | |
| --- | --- | --- |
|  | Univariate Cox regression | Multivariate Cox regression |
|  | HR (95%CI), P | HR (95%CI), P |
| Age |  |  |
| <65 | Reference |  |
| ≥65 | 0.60 (0.44, 0.81) <0.001 | 0.62 (0.45, 0.85) 0.003 |
| Body mass index | 1.04(0.96, 1.12), 0.360 |  |
| α-HBDH |  |  |
| ≤158 | Reference | Reference |
| >158 | 1.53 (1.13, 2.07) 0.006 | 1.36 (1.08, 1.80) 0.034 |
| LDH |  |  |
| ≤200 | Reference | Reference |
| >200 | 1.21 (1.04, 1.40) 0.036 | 0.98 (0.62, 1.48) 0.117 |
| Albumin |  |  |
| ≤40 | Reference | Reference |
| >40 | 0.96 (0.94, 0.99) 0.001 | 0.99 (0.96, 1.01) 0.263 |
| Gender |  |  |
| Female | Reference |  |
| Male | 0.98 (0.72, 1.32) 0.877 |  |
| Tumor location |  |  |
| Renal pelvis | Reference |  |
| Ureter | 1.20 (0.86, 1.69) 0.285 |  |
| Both | 1.35 (0.90, 2.05) 0.151 |  |
| Tumor size |  |  |
| <3 | Reference |  |
| ≥3 | 1.95 (1.38, 2.76) <0.001 |  |
| Tumor stage |  |  |
| ≤pT2 | Reference | Reference |
| >pT2 | 3.86 (2.72, 5.46) <0.001 | 2.42 (1.62, 3.61) <0.001 |
| Tumor grade |  |  |
| Low | Reference | Reference |
| High | 2.93 (1.78, 4.84) <0.001 | 1.98 (1.17, 3.37) 0.012 |
| Lymph node status |  |  |
| pN0/x | Reference | Reference |
| pN+ | 3.90 (2.76, 5.51) <0.001 | 2.17 (1.47, 3.19) <0.001 |
| Lymphovascular invasion |  |  |
| No | Reference | Reference |
| Yes | 2.17 (1.54, 3.07) <0.001 | 1.09 (0.74, 1.61) 0.647 |
| Tumor architecture |  |  |
| Sessile | Reference | Reference |
| Papillary | 1.44 (1.05, 1.97) 0.023 | 1.12 (0.80, 1.56) 0.507 |
| Surgery margin |  |  |
| Negative | Reference | Reference |
| Positive | 1.58 (0.94, 2.64) 0.084 | 0.98 (0.57, 1.69) 0.941 |
| Tumor necrosis |  |  |
| No | Reference |  |
| Yes | 1.41 (0.87, 2.27) 0.159 |  |
| Adjuvant systemic chemotherapy |  |  |
| No | Reference | Reference |
| Yes | 1.98 (1.45, 2.47) 0.012 | 1.29 (0.91, 1.86) 0.154 |

^a^All variables whose P values were less than 0.1 in the univariate Cox regression were included in the multivariate Cox regression.

^b^Abbreviations: HR=hazard ratio, CI=confidence interval, α-HBDH=α-hydroxybutyrate dehydrogenase, LDH=lactate Dehydrogenase.

**Supplementary Table 2.** The results of univariate and multivariate analysis about α-HBDH on cancer-specific survival in the group of patients with pT≤2.

| Variables | Cancer-specific survival | |
| --- | --- | --- |
|  | Univariate Cox regression | Multivariate Cox regression |
|  | HR (95%CI), P | HR (95%CI), P |
| Age |  |  |
| <65 | Reference |  |
| ≥65 | 0.66 (0.35, 1.24) 0.199 |  |
| Body mass index | 1.08 (0.92, 1.27) 0.356 |  |
| α-HBDH |  |  |
| ≤158 | Reference | Reference |
| >158 | 1.92 (1.05, 3.51) 0.033 | 2.04 (1.11, 3.74) 0.021 |
| LDH |  |  |
| ≤200 | Reference |  |
| >200 | 1.09 (0.58, 2.07) 0.786 |  |
| Albumin |  |  |
| ≤40 | Reference | Reference |
| >40 | 0.97 (0.95, 0.999) | 0.99 (0.96, 1.02) 0.610 |
| Gender |  |  |
| Female | Reference |  |
| Male | 1.09 (0.60, 1.98) 0.777 |  |
| Tumor location |  |  |
| Renal pelvis | Reference | Reference |
| Ureter | 2.39 (1.18, 4.82) 0.015 | 2.57 (1.27, 5.22) 0.009 |
| Both | 2.59 (1.18, 5.72) 0.018 | 2.35 (1.06, 5.20) 0.035 |
| Tumor size |  |  |
| <3 | Reference |  |
| ≥3 | 0.78 (0.43, 1.42) 0.414 |  |
| Tumor grade |  |  |
| Low | Reference |  |
| High | 1.58 (0.80, 3.14) 0.192 |  |
| Lymph node status |  |  |
| pN0/x | Reference | Reference |
| pN+ | 6.03 (1.86, 19.55) 0.003 | 7.03 (2.11, 23.39) 0.002 |
| Lymphovascular invasion |  |  |
| No | Reference |  |
| Yes | 1.32 (0.41, 4.28) 0.640 |  |
| Tumor architecture |  |  |
| Sessile | Reference | Reference |
| Papillary | 1.68 (0.90, 3.13) 0.098 | 1.09 (0.78, 1.59) 0.589 |
| Surgery margin |  |  |
| Negative | Reference |  |
| Positive | 1.35 (0.33, 5.60) 0.676 |  |
| Tumor necrosis |  |  |
| No | Reference |  |
| Yes | 1.40 (0.43, 4.52) 0.576 |  |
| Adjuvant systemic chemotherapy |  |  |
| No | Reference |  |
| Yes | 1.47 (0.88, 1.87) 0.104 |  |

^a^All variables whose P values were less than 0.1 in the univariate Cox regression were included in the multivariate Cox regression.

^b^Abbreviations: HR=hazard ratio, CI=confidence interval, α-HBDH=α-hydroxybutyrate dehydrogenase, LDH=lactate Dehydrogenase.

**Supplementary Table 3.** The results of univariate and multivariate analysis about α-HBDH on cancer-specific survival in the group of patients with pT>2.

| Variables | Cancer-specific survival | |
| --- | --- | --- |
|  | Univariate Cox regression | Multivariate Cox regression |
|  | HR (95%CI), P | HR (95%CI), P |
| Age |  |  |
| <65 | Reference | Reference |
| ≥65 | 0.73 (0.51, 1.03) 0.0722 | 0.64 (0.45, 0.91) 0.012 |
| Body mass index |  |  |
| α-HBDH |  |  |
| ≤158 | Reference | Reference |
| >158 | 1.41 (0.99, 2.01) 0.059 | 1.00 (0.62, 1.61) 0.952 |
| LDH |  |  |
| ≤200 | Reference | Reference |
| >200 | 1.28 (1.01, 1.67) 0.049 | 1.21 (0.92, 1.67) 0.098 |
| Albumin |  |  |
| ≤40 | Reference |  |
| >40 | 0.99 (0.96, 1.01) 0.292 |  |
| Gender |  |  |
| Female | Reference |  |
| Male | 1.03 (0.72, 1.46) 0.882 |  |
| Tumor location |  |  |
| Renal pelvis | Reference |  |
| Ureter | 1.10 (0.73, 1.65) 0.650 |  |
| Both | 1.31 (0.79, 2.17) 0.297 |  |
| Tumor size |  |  |
| <3 | Reference | Reference |
| ≥3 | 2.20 (1.33, 3.62) 0.002 | 2.12 (1.28, 3.52) 0.003 |
| Tumor grade |  |  |
| Low | Reference | Reference |
| High | 2.54 (1.12, 5.76) 0.026 | 2.67 (1.14, 6.25) 0.026 |
| Lymph node status |  |  |
| pN0/x | Reference | Reference |
| pN+ | 2.30 (1.58, 3.35) <0.001 | 2.02 (1.35, 3.02) <0.001 |
| Lymphovascular invasion |  |  |
| No | Reference | Reference |
| Yes | 1.48 (1.02, 2.15) 0.039 | 1.11 (0.75, 1.65) 0.604 |
| Tumor architecture |  |  |
| Sessile | Reference |  |
| Papillary | 1.00 (0.69, 1.45) 0.991 |  |
| Surgery margin |  |  |
| Negative | Reference |  |
| Positive | 1.17 (0.67, 2.04) 0.576 |  |
| Tumor necrosis |  |  |
| No | Reference |  |
| Yes | 1.05 (0.62, 1.77) 0.855 |  |
| Adjuvant systemic chemotherapy |  |  |
| No | Reference | Reference |
| Yes | 2.11 (1.76, 2.60) 0.007 | 1.84 (1.56, 2.32) 0.024 |

^a^All variables whose P values were less than 0.1 in the univariate Cox regression were included in the multivariate Cox regression.

^b^Abbreviations: HR=hazard ratio, CI=confidence interval, α-HBDH=α-hydroxybutyrate dehydrogenase, LDH=lactate Dehydrogenase.


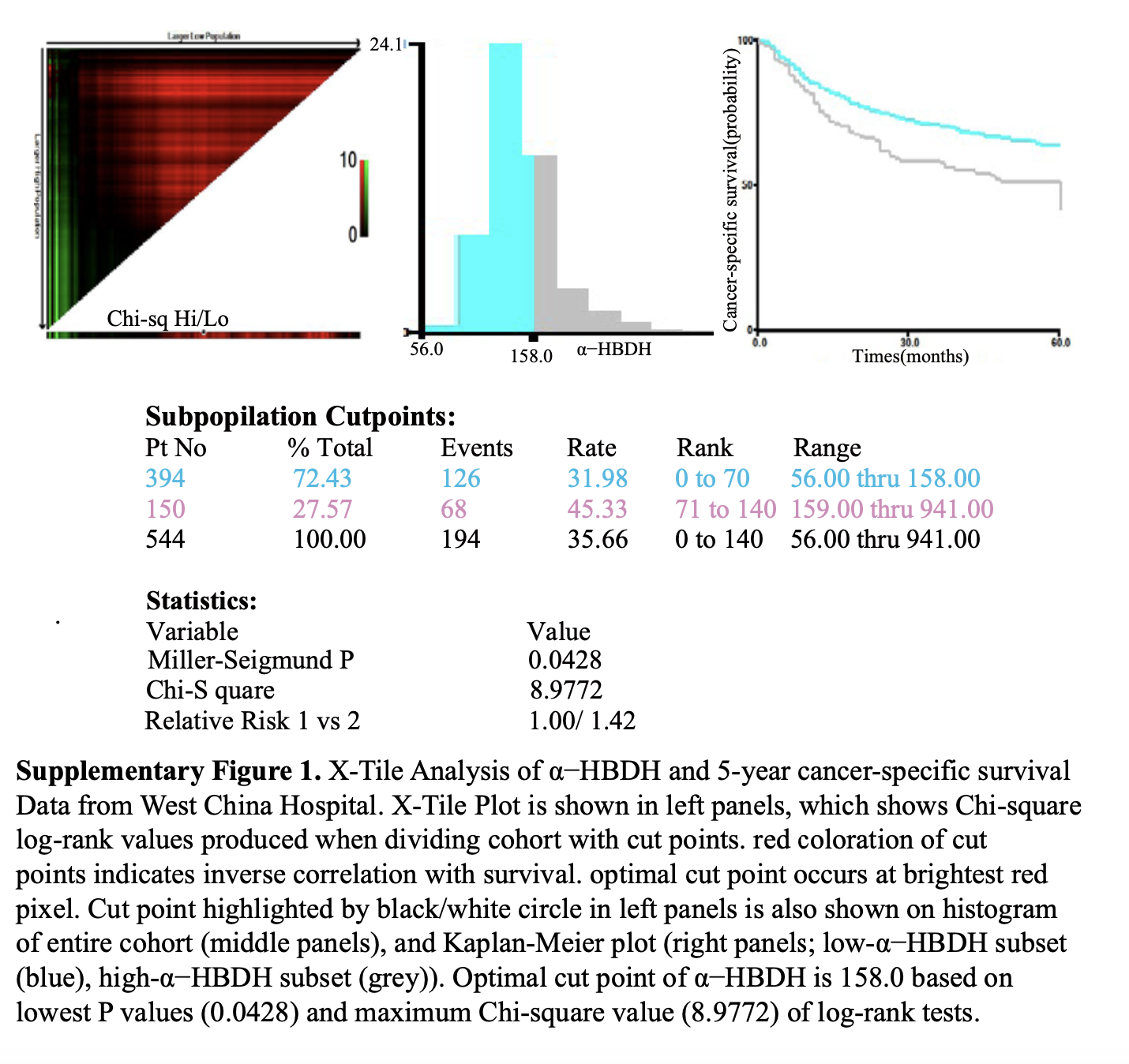


Supplementary Figure 1. X-Tile Analysis of α−HBDH and 5-year cancer-specific survival Data from West China Hospital. X-Tile Plot is shown in left panels, which shows Chi-square log-rank values produced when dividing cohort with cut points. Red coloration of cut

points indicate inverse correlation with survival. Optimal cut point occurs at brightest red pixel. Cut point highlighted by black/white circle in left panels is also shown on histogram of entire cohort (middle panels), and Kaplan-Meier plot (right panels; low-α−HBDH subset (blue), high-α−HBDH subset (grey)). Optimal cut point of α−HBDH is 158.0 based on lowest P values (0.0428) and maximum Chi-square value (8.9772) of log-rank tests.
